# Educational effects of and satisfaction with mixed-reality-based major trauma care simulator: A preliminary evaluation

**DOI:** 10.1097/MD.0000000000036816

**Published:** 2024-01-05

**Authors:** Han-Dong Lee, Yo Huh, Sora Kim, Ji-Woong Baek, Hojun Lee, Sang-Min Park, Jin-Kak Kim

**Affiliations:** a Department of Orthopedic Surgery, Ajou University, School of Medicine, Suwon, Republic of Korea; b Division of Trauma Surgery, Department of Surgery, Ajou University School of Medicine, Suwon, Republic of Korea; c Gyeonggi South Regional Trauma Center, Ajou University Hospital, Suwon, Republic of Korea; d Armed Forces Trauma Center, Armed Forces Capital Hospital, Seongnam, Republic of Korea; e Department of Orthopedic Surgery, Seoul National University Bundang Hospital, Seongnam, Republic of Korea.

**Keywords:** augmented reality, educational effect, extended reality, mixed reality, trauma care

## Abstract

Mixed reality (MR) is a hybrid system that projects virtual elements into reality. MR technology provides immersive learning using various real-world tools. However, studies on educational programs using MR are scarce. This study aimed to investigate the educational effects of and satisfaction with an MR-based trauma decision-making simulator. A total 40 of trainees self-selected to participate in this study. All of them participated in the MR trauma simulator for approximately 30 minutes and conducted voluntary learning without any external help. Declarative knowledge, measured using 20 multiple-choice questions, was assessed before and after MR trauma training. To confirm the educational effect, test scores before and after MR trauma training were compared using a paired *t*-test. Student satisfaction after training was measured using a ten-item questionnaire rated on a five-point Likert scale. A pretest–posttest comparison yielded a significant increase in declarative knowledge. The percentage of correct answers to multiple choice questions increased (from a mean of 42.3, SD 12.4–54.8, SD 13) after the MR-based trauma assessment and treatment training (*P* < .001). Of the participants, 79.45% were satisfied with the overall experience of using the MR simulator. This study demonstrated a meaningful educational effect of the MR-based trauma training system even after a short training time.

## 1. Introduction

Extended reality (XR)-based technologies open novel ways of teaching and training for medical education, as they allow for immersive experiences. XR technology is subdivided into virtual reality (VR), augmented reality (AR), and mixed reality (MR).^[1–3]^ It is classified into whether the head-mount-display (HMD) that provides the image delivers only complete digital information (VR) or whether virtual information is overlaid on real information through translucent glass (AR, MR). In addition, virtual information can be directly manipulated by the user (VR, MR), or information can be provided unilaterally (AR). MR can manipulate virtual information in the same manner as VR, and, as in AR, virtual information can be added to real information to give a more immersive feeling.

Trauma is one of the leading causes of death among young people worldwide.^[4–7]^ Among trauma patients, 50% of deaths occur within the first hour and 30% within a few hours.^[8]^ High proficiencies are required for rapid treatment to reduce preventable mortality. Therefore, trauma care education is highly important.^[9]^ However, the current trauma education system is limited in the treatment process and training, as the patient’s condition is critical and treatment is urgent. Moreover, there are not many trauma centers that have implemented such treatment.^[10]^ Although it is replaced by lecture education or practice using a theatrical simulation, it is somewhat different from the actual treatment environment; therefore, it is difficult to adapt immediately to an urgent situation.^[10,11]^

For more immersive education, various technologies using VR for trauma patient treatment have been developed, and satisfaction with and immersion in VR-based education were higher than those of lectures or mannequins.^[4,12–18]^ However, VR-based education is limited to virtual information and interaction with only the controller; therefore, most of the training was to triage the patients in a mass casualty situation.^[16–19]^ MR technology enables more immersion in the treatment of trauma patients through the mixing of real and virtual information and helps use various medical tools used in the actual trauma patient treatment process to help students learn more efficiently.^[20]^ However, MR equipment is not widely distributed yet; therefore, the development of medical education programs using it is limited.^[1]^ Although there are reports of education on the treatment of trauma patients using MR technology, there are cases where it was developed with the goal of learning specific skills rather than the entire process of treating trauma patients.^[21,22]^

This study aimed to examine the educational effect and satisfaction of a tool to learn the entire process of actual trauma patient treatment using MR technology.

## 2. Methods

### 2.1. Ethics approval

This study was approved by the institutional review board of Ajou University Medical Center (AJOUIRB-SUR-2021-437) and Seoul National University Bundang Hospital (B-2011-649-101).

### 2.2. Participants

Open recruitment for this study was conducted using posters in one level-one trauma center, one medical school, and one nursing college. The research information, including the objectives, inclusion criteria, and exclusion criteria of our study, was advertised through notices posted in hospitals and on campus bulletin boards. The inclusion criteria were adults who were interested in participating in trauma education training with XR, including trauma specialist nurses, nursing students, and medical students. The exclusion criteria were minors under the age of 19, individuals who previously received trauma training using MR, individuals who had side effects from the use of MR-related equipment, individuals who had a history of epilepsy, pregnant, individuals who had heart or brain organic diseases, individuals who had psychiatric problems, individuals who had a hearing or vision impairment, and individuals who had a history of severe motion sickness. Participants from each institution voluntarily participated in the trial and were provided with information about the study. Informed written consent for the study and the publication was obtained before the simulation. Before conducting the study, information on age, gender, educational background or work experience, and VR and MR experience was collected. VR and MR experiences were evaluated using an eight-item questionnaire rated on a five-point Likert scale based on a previous study.^[23]^ As the participants were vulnerable subjects, all personal information was blinded to avoid being exposed to the research director.

### 2.3. Description of the MR-based major trauma care system

**Figure 1.**
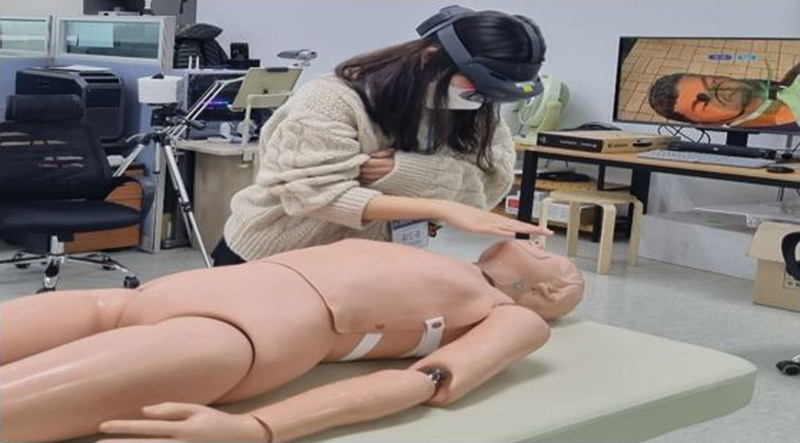
Photo of real training scenes wearing HoloLens 2.

**Figure 2.**
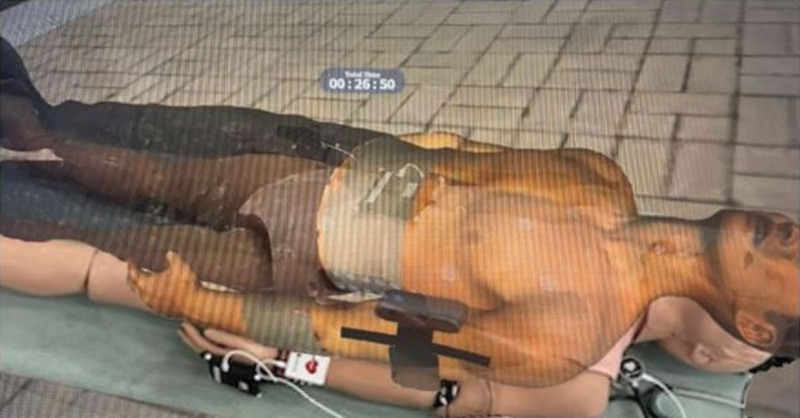
Screenshot of a virtual patient overlaid on a mannequin.

**Figure 3.**
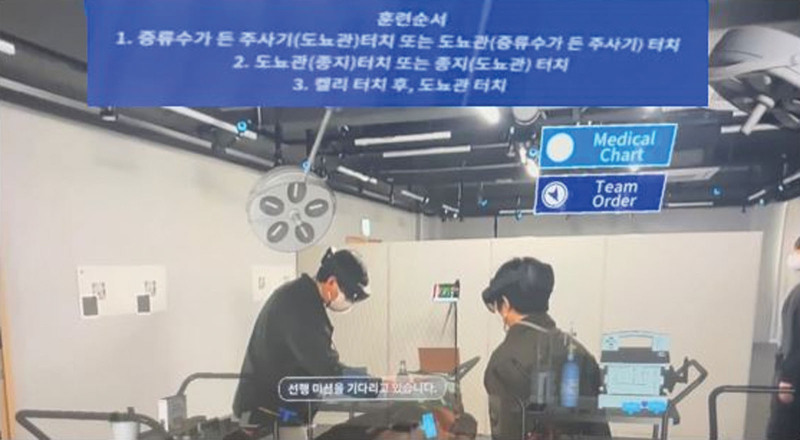
Screenshot showing the digital mentor instructing a trainee on the appropriate treatment via a text box.

**Figure 4.**
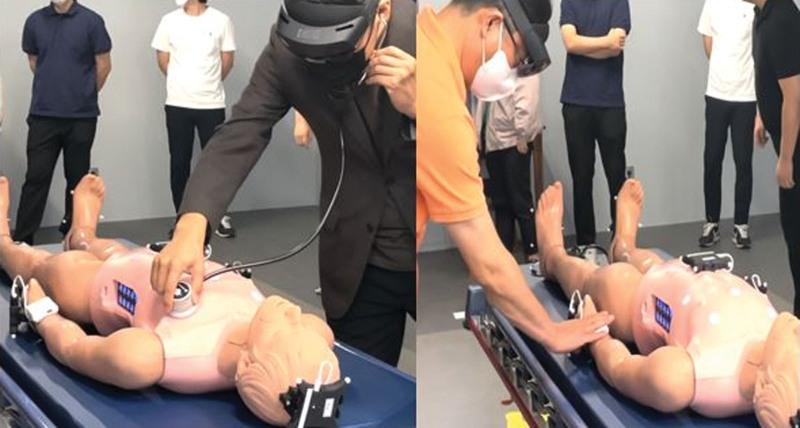
Photo of Stethoscope and pulse band for MR. MR = mixed reality.

**Figure 5.**
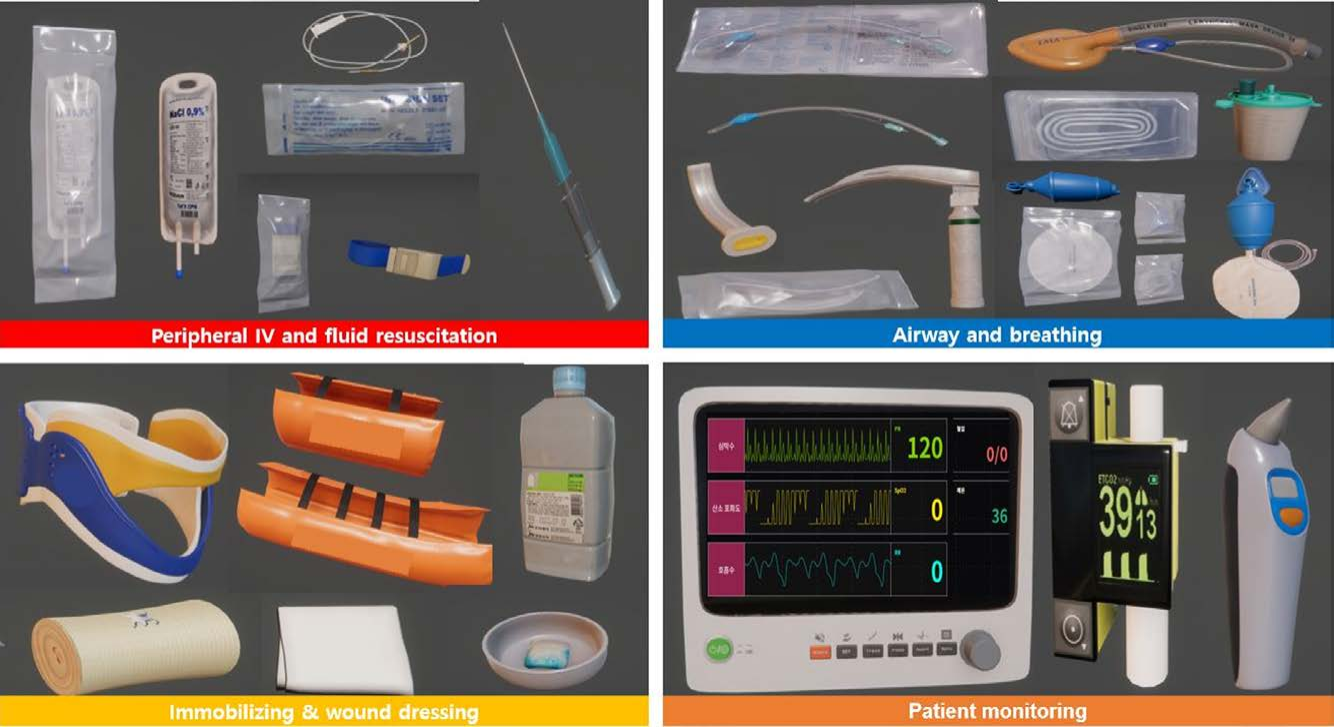
Virtual medical equipment and items for performing the ABCDE procedure for the initial treatment of trauma patients. ABCDE = airway, breathing, circulation, disability, and environment.

Trauma Nursing XR training course (TNXRtc, DKI Technology, Republic of Korea, Seoul) is an MR-based first aid training system for patients with severe trauma using Holones 2 HMD (Microsoft Corp, Redmond, WA) (Fig. 1). On the HMD screen, a virtual patient (medical twin) is overlaid on a prefixed mannequin (Fig. 2). The virtual patients are medical twins based on real patients, and medical twins were formed using paramedic notes, medical records, vital signs, labs, X-ray, and CT images, and pictures of affected areas of patients from 3 level-one trauma centers. The digital mentor teaches how to assess and treat virtual patients according to the catastrophic hemorrhage, airway, breathing, circulation, disability, and environment process through a popup textbox, and the trainees learn it independently (Fig. 3). There are various patient evaluation methods that can be performed in the TNXRtc environment. Basic assessment of the patient is possible by tapping the virtual patient’s shoulder to confirm consciousness, examining the chest and abdomen and affected areas, auscultation of the chest and abdomen using an MR stethoscope, and pulse palpation using an MR pulse band (Fig. 4). Participants can check the patient’s vital signs by attaching a virtual monitoring device to the patient, pupil dilation by a virtual pen light to check, and the body temperature with a virtual thermometer. Treatment of the patient can include dressing and splint attachment to the affected limb and supplying fluid for massive bleeding. Basic patient care, such as securing an airway and wearing a cervical orthosis, attaching an oxygen mask and adjusting oxygen volume, thoracic needle decompression, and cricothyroidotomy can be conducted (Fig. 5).

Depending on whether the trainee is performing the appropriate treatment within a certain period of time, the virtual patient’s status changes to be more immersive and to increase the learning effect. Changes in the patient’s status include changes in the virtual patient’s consciousness, respiration, and bleeding volume in the virtual patient projected on the HMD, as well as changes in vital signs on the virtual monitor. Feedback on changes in the patient’s condition is provided by a stethoscope or pulse band developed for MR.

TNXRtc includes scenarios of patients with open fractures of the extremity, amputation, penetrating and crushing injuries of the abdomen and thorax, and brain and spinal cord injuries. Each class was prepared to last approximately 30 minutes. In this study, a scenario was used to learn the on-site assessment and treatment of severe trauma patients with open limb fractures during the TNXRtc procedure. The scenario is written based on Advanced Trauma Life Support 10th Edition, Trauma Nurse Core Course 8th Edition, and Korean Trauma Assessment and Treatment 2nd Edition. In TNXRtc, a maximum of 5 people can be trained under the leadership of a team leader; however, in this study, a scenario in which one individual assesses and treats was used.

Prior to the TNXRtc class, approximately 30 minutes of pretraining was performed for all participants. First, it was explained that this class was intended to learn the ABCDE sequence of assessment and treatment for patients with upper and lower extremity open complex fractures. The proper way to wear the HoloLens (the touch method using hands) and the examination method was taught. Interfaces, such as the registration method for class participation and the operation method for virtual tutors, were also explained. Moreover, the virtual and MR tools at the treatment site were briefly overviewed.

### 2.4. Evaluation of outcome (Fig. 6)

A gain in declarative knowledge was determined by asking 20 multiple-choice questions before and immediately after working with TNXRtc. The questions were composed of information necessary for the initial treatment of major trauma patients with open fractures. The questions inquired regarding the sequence of appropriate diagnosis and treatment based on the ABCDE sequence, appropriate diagnostic methods, such as the location of pulse confirmation according to the location of the extremity fracture, tools necessary for each stage of treatment, such as the size of the appropriate needle for the intravenous route, and appropriate treatment methods, such as the appropriate amount of fluid to be administered during fluid therapy. The questions were constructed based on the ATLS guideline. The total score was calculated as 5 points for each of the 20 items and converted into a total of 100 points. Influence of preexisting knowledge and gain in knowledge were measured by subgroup analysis with regard to baseline demographic factors.

**Figure 6.**
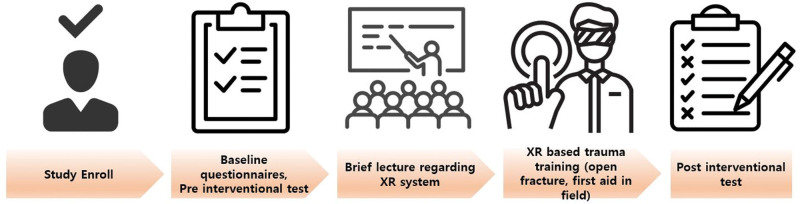
Test process to confirm declarative knowledge and satisfaction.

The second outcome was the time it took to complete the MR class. Relevance with age, gender, occupational group, and VR experience was analyzed using subgroup analysis.

The third outcome was satisfactory with the MR education for trauma care. Participants rated ten statements to measure the satisfaction of TNXRtc, which consisted of 5 items: similarity to reality, help in learning, enjoyment, satisfaction compared to existing teaching methods, and immersion. Each item consisted of 2 questions. Participants rated these aspects on a five-point Likert scale ranging from 1 (fully unsatisfied) to 5 (fully satisfied). Total satisfaction was calculated by converting the total score of each question to 100%. The correlation with each underlying factor for satisfaction was investigated.

### 2.5. Statistical analysis

To confirm the educational effect, test scores before and after treatment were compared by paired *t*-test. Chi-squared test and Pearson correlation analysis were performed to find factors related to the level of improvement in score, time taken for class, and level of satisfaction depending on the type of variables. Data were analyzed with SPSS version 20.0 (IBM, Armonk, NY). In all analyses, *P* < .05 was taken to indicate statistical significance.

## 3. Results

A total of 40 participants were enrolled. Seven participants were trauma nurses, 19 participants were medical students, and 14 participants were nursing students. Of the 40 participants, 25 (62.5%) were men and 15 (37.5%) were women. The mean age was 24.6 ± 4.5 years (range: 19–39 years). Seven were trauma nurses, and the average working experience was about 9.9 ± 5.0 (range: 4–17 years). There were 14 nursing students, including 5 3rd graders and 9 4th graders. There were 19 medical students, including 5, one, 6, and 7 students in the 2nd, 3rd, and 4th grades, respectively.

**Figure 7.**
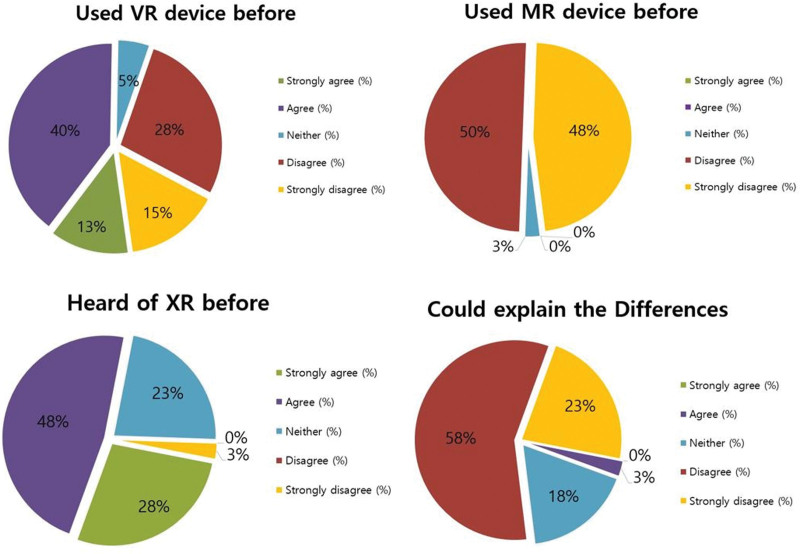
Participants’ XR experience. XR = extended reality.

Regarding the experience with XR, 30 participants (75%) had heard of XR before, whereas 32 participants (81%) could not explain the difference between XR technologies. Twenty-one participants (51.2%) had experience with the VR device before, and no participant had experience with the MR device (Fig. 7).

It took an average of 29.5 ± 7.3 minutes to perform self-learning using MR (range: 15.8–50.0 minutes). In correlation analysis, MR learning time was longer among women (*R* = 0.617, *P* = .016). In addition, the MR learning time increased with increasing age (*R* = 0.358, *P* = .023) and less XR experience (*R* = 0.324, *P* = .041).

**Figure 8.**
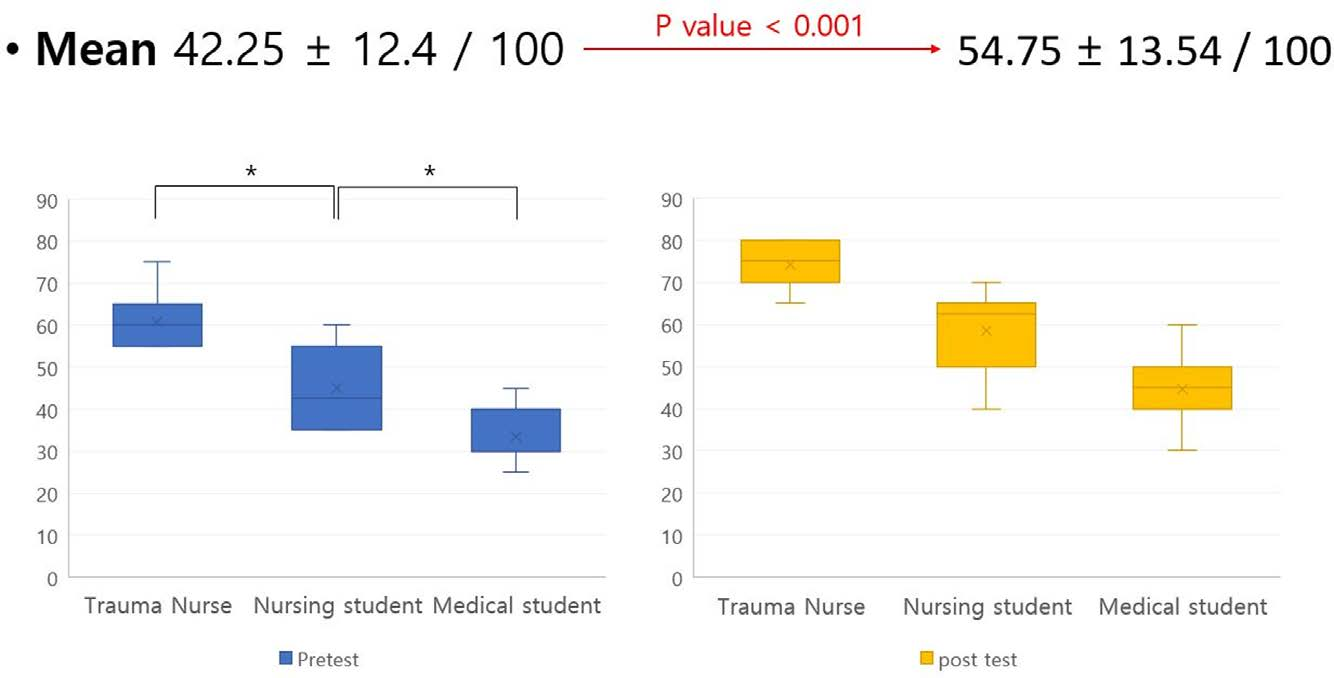
The effectiveness of acquiring declarative knowledge using MR trauma simulator. MR = mixed reality.

The test score before MR learning was 42.3 ± 12.4 (range: 25–75) and increased to 54.8 ± 13.5 (range:30–80) after MR learning, which was statistically significant (*P* < .001; Fig. 8). Pretest scores were highest in trauma nurses (60.7 ± 7.3), followed by nursing students (45.0 ± 9.4) and medical students (33.4 ± 5.5; all *P* < .001). Posttest scores were also highest in trauma nurses (74.3 ± 6.1), followed by nursing students (58.6 ± 9.5) and medical students (44.7 ± 7.4; all *P* < .001) The degree of elevation of the score was not correlated with any baseline demographic factor or XR experience (all *P* > .05).

**Figure 9.**
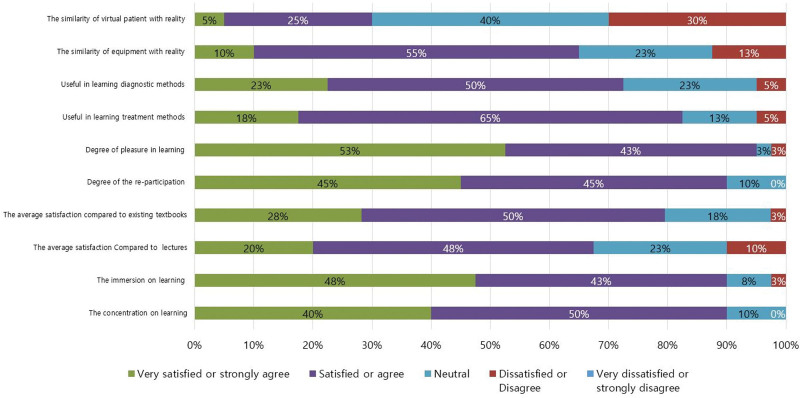
Results of the participants’ satisfaction with the MR trauma simulator. MR = mixed reality.

Figure 9 shows the results of participants’ satisfaction with the MR trauma simulator. Overall satisfaction was 79.45%. The average level of satisfaction with the similarity of patients and equipment with reality was 3.1 and 3.6, respectively. The average scores for being useful in learning diagnostic methods and treatment methods were 3.9 and 4.0, respectively. The degrees of pleasure in learning and the re-participation rate were 4.5 and 4.4, respectively. Compared to existing textbooks and lectures, the average satisfaction scores were 4.0 and 3.8, respectively. The immersion and concentration on learning were 4.4 and 4.3, respectively. Table 1 shows the contents of the questionnaire and responses. Overall satisfaction was significantly higher among younger participants (r = -0.271, *P* = .021) and students (*P* = .011). Satisfaction with learning about treatment methods was higher in men (*P* = .027). Satisfaction was not related to the degree of XR experience (all *P* > .05).

**Table 1:** Questionnaire contents and participant responses.

Qusetionnaire item	Very dissatisfied or strongly disagree, n (%)			Dissatisfied or disagree, n (%)			Neutral, n (%)			Satisfied or agree, n (%)			Very satisfied or strongly agree, n (%)		
	Trauma nurse	Nursing student	Medical student	Trauma nurse	Nursing student	Medical student	Trauma nurse	Nursing student	Medical student	Trauma nurse	Nursing student	Medical student	Trauma nurse	Nursing student	Medical student
The similarity of virtual patient with reality	0(0)	0(0)	0(0)	2(5)	2(5)	8(20)	4(10)	6(15)	6(15)	1(2.5)	5(12.5)	4(10)	0(0)	1(2.5)	1(2.5)
The similarity of equipment with reality	0(0)	0(0)	0(0)	1(2.5)	2(5)	2(5)	4(10)	1(2.5)	4(10)	2(5)	9(22.5)	11(27.5)	0(0)	2(5)	2(5)
Useful in learning diagnostic methods	0(0)	0(0)	0(0)	1(2.5)	0(0)	1(2.5)	5(12.5)	0(0)	4(10)	1(2.5)	8(20)	11(27.5)	0(0)	6(15)	3(7.5)
Useful in learning treatment methods	0(0)	0(0)	0(0)	2(5)	0(0)	0(0)	4(10)	0(0)	1(2.5)	1(2.5)	10(25)	15(37.5)	0(0)	4(10)	3(7.5)
Degree of pleasure in learning	0(0)	0(0)	0(0)	1(2.5)	0(0)	0(0)	1(2.5)	0(0)	0(0)	3(7.5)	2(5)	12(30)	2(5)	12(30)	7(17.5)
Degree of the re-participation	0(0)	0(0)	0(0)	0(0)	0(0)	0(0)	1(2.5)	0(0)	3(7.5)	5(12.5)	4(10)	9(22.5)	1(2.5)	10(25)	7(17.5)
The average satisfaction compared to existing textbooks	1(2.5)	0(0)	0(0)	0(0)	1(2.5)	0(0)	2(5)	2(5)	3(7.5)	3(7.5)	6(15)	11(27.5)	1(2.5)	5(12.5)	5(12.5)
The average satisfaction compared to lectures	0(0)	0(0)	0(0)	2(5)	0(0)	2(5)	2(5)	2(5)	5(12.5)	2(5)	9(22.5)	8(20)	1(2.5)	3(7.5)	4(10)
The immersion on learning	0(0)	0(0)	0(0)	0(0)	0(0)	1(2.5)	0(0)	1(2.5)	2(5)	6(15)	3(7.5)	8(20)	1(2.5)	10(25)	8(20)
The concentration on learning	0(0)	0(0)	0(0)	0(0)	0(0)	0(0)	1(2.5)	1(2.5)	2(5)	4(10)	5(12.5)	11(27.5)	2(5)	8(20)	6(15)

## 4. Discussion

### 4.1. Principal findings

The main goal of this study was to test TNXRtc as an educational tool and determine its effect on knowledge gain and student satisfaction. All participants completed the MR-based self-learning trauma patient treatment class. It took slightly longer for women, older people, and people with less XR experience. A pretest-posttest comparison yielded a significant increase in declarative knowledge. The results indicated that the MR-based trauma patient treatment system had a learning effect on both experienced and inexperienced students in the emergency treatment of trauma patients. This was irrespective of age, gender, or experience with MR. Overall satisfaction was high, especially in younger students.

Since the trauma scene is highly urgent, the quality of highly trained medical attendants is crucial. Therefore, educating medical attendants on trauma care is important.^[9]^ However, as trauma treatment is critical for learners to participate in directly, educational opportunities are limited.^[10,11]^ Traditionally, theatrical practice using training mannequins was mainly performed. As mannequins cannot exhibit various traumatic effects, the level of immersion is low. To date, experts had to attend training; therefore, the consumption of human and material resources was necessary. In addition, trainees do not have sufficient time to repeat the procedures, and when they practice alone, it is difficult to assist them properly.^[24]^

Therefore, various educational programs using XR have been developed for the acute treatment of trauma patients. Initially, relatively simple education, such as patient classification in a mass disaster, was applied to VR.^[15–17,25]^ These studies have demonstrated that virtual reality can be more immersive and cost-effective in trauma patient care.^[16]^ Recently, it has been developed to learn not only the classification of patients but also the decision-making for the proper treatment sequence and treatment location needed to treat the actual patient based on the ATLS guideline.^[4,13]^ In addition, the HMD was used to increase the sense of immersion.^[18]^ Furthermore, VR education programs have been developed to enable self-learning through the development of algorithms.^[12]^

With the recent development of technology, the development of a training program for trauma patients using MR has been reported in several studies.^[21,22,26,27]^ Unlike VR, which is completely virtual, MR is a mixture of reality and virtual reality and can give trainees a more immersive feeling in reality.^[27,28]^ Moreover, it is possible to practice actions such as securing an IV line using an actual needle or performing chest compression directly on a mannequin, which can help form more muscle memory.^[21,22,26,27]^ The TNXRtc used in this study used MR and equipment similar to real tools, allowing learning to be more immersive. It was possible to examine using a visual recognition function and facilitate it using a hand recognition function. By using a stethoscope for MR, interaction with virtual information increased. In addition, a more realistic situation was realized through a haptic response using a pulse band for MR. TNXRtc differs from existing MR-based trauma training in that it can learn not only one technique but also the entire treatment of one virtual trauma patient.

However, MR technology has not yet been popularized, and many questions remain. First, there may be questions about whether the trainee can adapt well to such an environment. Devices such as Oculus Quest 2 (Oculus, Irvine) and Vive (HTC, Taiwan) became available for purchase by nonprofessionals, and as popularized VR game facilities appeared, many people came into contact with VR. In this study, approximately 50% of the participants said that they had experience with VR. However, no one had any experience with MR. Nevertheless, most of the trainees were able to adapt well to MR with only a short 30-minute pretraining, and most were able to fit the class within approximately 30 minutes. There were differences according to experience, gender, and age of XR. Therefore, it is necessary to consider that the class time may vary depending on trainee characteristics during MR education.

The improvement of knowledge by TNXRtc MR training was observed to be statistically significant even in a 30-minute single class. As in the previous XR-based medical education research, this study evaluated the educational effect through test items.^[1,29–33]^ A significant improvement in declarative knowledge was observed. This improvement in scores was also observed for experienced non-novice users. It was independent of age, gender, and XR experience. This suggests that MR education has the potential to show sufficient educational effects regardless of age, gender, or XR experience.

In a systematic review in the past, the satisfaction of education using XR was inconclusive.^[34]^ A recent study demonstrated high satisfaction due to easy use and high immersion.^[14,24,30,35]^ The overall satisfaction level of the MR-based TNXRtc system was similar to that of the previous MR system.^[24]^ The MR-based TNXRtc system received relatively high scores in terms of immersion and interest. However, it received relatively low scores in terms of similarity to reality. This is likely related to the spec of HoloLens2 HMD, a device for XR. Although the FOV of HoloLens2 has increased to 52 degrees compared to the previous work’s 30 degrees, there is still an empty space in the field of view; due to the limitations of CPU and resolution, it seems that there is a limit to express various traumatic items and urgent emergency situations.^[36,37]^

Little is known regarding the factors that influence the satisfaction of XR learning. In this study, satisfaction was high among younger participants and students. A previous medical study using VR reported that satisfaction was higher in younger grades.^[30]^ It is presumed that the younger the trainees are, the more likely they are to adapt to these skills and become more familiar with them.

## 5. Limitations

This study has some limitations. First, the results may have been influenced by the fact that participation in this study was on a voluntary basis and included mainly motivated students. As motivated students are more likely to participate in study, future studies should investigate if less motivated students could also benefit from playing TNXRtc. As a pilot study, no comparison with other teaching methods was presented. It is important to determine the value of MR-based education to evaluate whether there is an educational effect or higher satisfaction than with existing methods through a randomized study using traditional education methods, such as lectures and mannequins. The test used in this study to measure educational effectiveness has not been proven to be reliable. In future studies, the test questions should be revised and applied by confirming the reliability based on the results of this pilot study.

## 6. Conclusions

This study demonstrated a meaningful educational effect of and satisfaction with the MR-based trauma training system even after a short training time. Further research is required to compare the effectiveness of this method and traditional education methods, such as lectures and mannequin-based simulation.

## Author contributions

**Investigation:** Yo Huh, Sora Kim, Ji-Woong Baek, Hojun Lee.

**Writing – review & editing:** Han-Dong Lee, Sang-Min Park, Jin-Kak Kim.
